# Change is just more of the same

**DOI:** 10.2349/biij.4.4.e36

**Published:** 2008-10-01

**Authors:** BJJ Abdullah, KH Ng

**Affiliations:** 1 Department of Biomedical Imaging, University of Malaya, Kuala Lumpur, Malaysia

“Modern skulls still have a stone age mind” [1]

“This Quality Assurance programme is a total waste of time.”

“Why change the roster if it was working just fine before?”

“If it isn’t broken, why do you want to fix it?”

“The bosses never tell us what’s going on, but they expect us to follow them!”

“Why can’t they make up their minds about how they want to improve the standards in our academic department instead of changing things all the time?”

“It’s just another new Dean of the faculty who thinks he knows it all and wants to change things.”

“Don’t you remember the previous attempt by the Vice-Chancellor to change our university? It ended up in a total failure, didn’t it?”

“All this stuff about change management is an excuse for the consultants to make lots of money.”

“The only way we are going to survive is if we destroy everything and then, like the proverbial phoenix, we rise from the ashes.”

Type the word “change” into Google and you get 1.4 billion hits. “Change management” gets you 71.8 million hits, “coping with change” 2.6 million and “books on change” 302,000 hits. In short, change is BIG.

Think of a change that you recently experienced. Did you like that change? Or were you unhappy with it? Did you take a long time to come to terms with it, or was it a breeze? Or did you forget about it for another time and place? Were you able to stop the change? In the end, does it really matter whether you liked it or not? After all, it has already happened.

Change is all around us, and it is close to impossible to define because it can be seen from so many different perspectives. We find change disorienting as it fills us with an anxiety similar to the loss of something that had defined our lives. With an established routine, we don't have to think! And it is so easy to forget that thinking is really hard work, especially if everything needs to be thought out every day! It is akin to experiencing a “culture” shock where we experience the unease of a different culture with different customs, values, and language. This is due to the absence of the recognisable cues or signposts we took for granted in our “familiar” culture. Add death to the scene and change is never easy. The death of a relationship is almost worse than the physical death of a loved one.

Yet each of us craves change that brings benefits. Businesses want to be more profitable, more efficient, faster to market and more innovative. Athletes want to run faster, jump higher, throw further, score more goals and break records. Educators are always looking for more effective best practices and processes. Doctors are always looking for more effective treatments, better outcomes, less morbidity, faster reports, quicker scans and more resources to do be able to do more. Researchers are looking for the breakthroughs that will win them the Nobel Prize. Entrepreneurs are looking for the next big trend that will be the next iPod and make them millions. Children want to grow up and become adults sooner so that they can buy their Lamborghinis. In other words, most people want CHANGE when it is in their interests, the only caveat being that everything else stays the SAME!

Many people wish to freeze the present and hold things static in the mistaken belief that by resisting or ignoring change they can control the world around them. We wish to do our barium enemas the same way as we have always done; we do not want to learn about that new MR colonography, or get sucked into all the hype about PET/CT! Teleradiology is not my cup of tea as I have enough work already!

However almost everything we experience is alive: our ideas, our values, our passions, our families, our friends, our colleagues, and our communities. All these things need “movement” to continue to be alive. They change every day, very often in subtle ways that we do not notice, until one day we find that the ground under us has shifted. The reason we work is to create similar movements, to produce change for ourselves, our colleagues, our patients, our hospitals and our communities. Without movement towards some predetermined acceptable goal, life will be a drag and we will all suffocate. What are the consequences of inability to change? Entropy. Slow certain death.

At the same time, we cannot steamroll blindly towards change. Contrary to the emphasis on change, the importance of stability amidst all the change is very often overlooked. Without a certain degree of stability, most things would exist for no longer than a split second. Without specialities in medicine, healthcare would be a free-for-all which would make life for patients a total chaos. Conversely without a certain amount of continuous change, things would remain the same forever. For instance, there would be no MR guided ultrasound which transcends imaging, intervention and surgery or targeted or chemotherapy.

Stability and change complement each other and should be treated as interdependent conditions. Both of these states existing alone raise serious problems. Excessive change leads to chaos while too much stability results in inertia. However, when we put stability and change together, possibilities open up. Without the continuity provided by stability – without connections between the past, present, and future – and disruption provided by change, there would be no reason to grow, learn or have dreams. Change only benefits an individual, a family, an organisation or a community if stability is also part of the change. Indeed, these connections provide the basis for trust, durable social relationships, the rule of law, and robust communities. Stability is the keystone of human identity [2].

Unfortunately stability is often perceived as a lack of change or even as resistance to change. Change, on the other hand, is always perceived as positive and to be pursued. It is no wonder that the call for change is a top priority every time a failure occurs or a new challenge comes along. Suggestions for change are easily funded and readily implemented but often with poor results. This tells us that we should not view stability in the same way as stasis or paralysis.

Stable systems, contrary to common belief, must be highly adaptive and flexible. This is exemplified by our human body and homeostasis. Homeostasis is the property of open or closed systems in an organism that regulate its internal environment so as to maintain a stable, constant condition. Multiple interacting dynamic adjustments and feedback mechanisms make homeostasis possible. However when too many of its systems collapse, the organism is unable to maintain itself and succumbs. Therefore systems must have the capacity to change in order for it to stay the same. The challenge, albeit easier said than done, is finding the right balance between stability and change; the “yin” and the “yang”.

So, why is the change process so scary? Because change upsets people. It changes the goal posts, changes the rules we have become so familiar with. It disrupts our routine and habits. It can be very unnerving when the familiar packaging of your favourite coffee is changed, or when someone takes “your” seat on the restaurant, train or bus or even when “your” toilet at work is occupied. Change demands constant adaptation. But imagine everything changing at the same time: how you work, what you work on, the principles on which your work is based, your work rules, how success is measured, what is socially acceptable, the cost of living, the way of living – this is change multiplied manyfold. Even though change is inevitable and essential the acceleration of the pace of change is frightening. But while we may not be able to control change, we can certainly control our attitude towards change.

**Figure 1:**
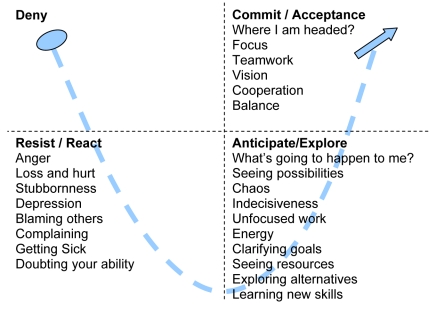
Phases of going through change.

We tend to respond to change the same way we respond to anything we perceive as a threat. We go through denial-resistance-anticipation / exploration- commitment (Figure 1). There are those who categorise people in the change process zoologically [3], for example:

- Ostriches - They work on the principle that if they cannot see the change then it’s not there. They seal themselves off from those around them, i.e. they cocoon themselves and try to ignore what is happening.
- Moles – They disappear when change is going on and then they pop up when they think everything has been completed.
- Tigers – They fight tooth and claw all the way in the change process. They are extremely sensitive and if you hurt them only a little, they will seek to hurt you a whole lot more. Their motto is “Go make your change elsewhere with little people but don’t mess with us!”
- Dogs – They tend to be more powerful in a pack. They seek one another out and attack en masse. They are not fearless but they know that together they create even more fear and damage.
- Owls – These people know better than you and are not slow to point this out. They enjoy pointing out all the little faults in your change project which for them is below their level. They ooze negativity, destructive criticism, and even conduct plain sabotage.
- Snails - They go so slowly that change does not really affect them, since by the time they get to their destination, the posts may have shifted somewhere else. Basically they hope that you will leave them to their own devices.

Do any of your colleagues fit any of the above descriptions?

When we are not comfortable with change, we often rationalise why the proposed change is bad. We wish to stay where we are because we feel that our needs have already been met; we justify it by saying that we have invested heavily to get where we are or that we are in the middle of something important. Alternatively we do not trust the person driving the change, we think that the proposed destination or journey is not right and looks worse than where we are now, too much relearning is required, or there is nothing to attract us forward. The more horrifying the scenario, the more likely we are to believe in the reasons not to change, e.g. the end of a way of life, the veste    d interests behind the change, the loss of some moral high ground, the end of a speciality, or the loss of “territory”. This becomes a rallying cry for different groups of people who have a variety of reasons to resist change, which often have little to do with the change itself. But in resisting change, we forget how we arrived at the current state that we consider to be sacrosanct – it evolved from some other state that those before us had resisted too!

What about doctors? How well do they cope with change? Are we doing a good job of changing? The answer is: not really. A physician’s background, ethics, and beliefs strongly mould his or her opinion and influence his or her practice behaviour. As human beings, physicians are motivated by multiple interests: the patient’s interests, their own interests, society’s interests, and, increasingly, the payor’s interests. Physicians must balance their multiple motivations with a professional ethos that demands accountability; competence, if not perfect performance; willingness to admit mistakes that occur; maintenance of requisite knowledge and skills; and willingness to admit ignorance and ask for help. These special features of a physician’s background makes practice behavioural changes very complex [4]. New diagnostic techniques and advanced therapies are vital in improving the quality of healthcare but these technologies, in themselves, are insufficient.

Traditional approaches to address physicians’ lack of awareness and lack of familiarity, such as continuing medical education and dissemination of evidence-based guidelines, have proven ineffective in changing practice behaviour [5]. Generally single interventions, such as educational materials, reminder systems, audit and feedback, have modest or almost negligible effects when used alone. However, the use of combined intervention strategies can result in significant changes in physician behaviour and improved health outcomes [6]. In a cohesive, balanced approach to planning successful interventions for improving practice, behavioural theories must be supported with consideration of the organisational dimension. Any intervention designed to have an impact on behaviour must be considered from a multidimensional perspective.

Whose responsibility is it to manage change? Is it the heads of government or ministers, the industry, the leaders of organisations, professors, professionals, the staff, parents, or the institutions of learning like schools and universities? As much as we would like to transfer responsibility for change to others, everyone must be part of the solution. Leaders have a greater responsibility to facilitate and enable change (not to instruct and impose) especially to understand the situation from an objective standpoint and help employees understand why, how and when to respond positively depending on individual situations and capabilities. Managing the change process is no easy task! The best organisations learn externally as well as internally, and successful adjustment to change is not just movement, but movement with predictability.

There have been numerous management tools which have been formulated by management gurus with their “theory of the decade”, to assist in managing change in organisations (Table 1). The complexity of some of these constructs makes any sensible use impossible. The tendency is to apply one of these techniques over a time frame and expect change to happen – a one-size-fits-all approach that often backfires [7]. Change is a process and not a one-off event or even a series of events. Even though events help to focus people’s attention, they are only one part of the change equation. It is the ongoing practice that enables long-term success.

**Table 1:** The numerous tools for change

Globalization Information Technology Total Quality Benchmarking Best Practices Customer Focused Micromarketing Outsourcing Flexible Manufacturing Value Creation Core Competence Partnering Competitive Advantage Networks Strategic Alliances Concurrent Engineering Delaying Information Revitalization Computer-aided Design	Computer-aided Engineering Mission Cross-functional Teams The New Organization Diversity Empowerment The Information Organization The Hybrid Organization Knowledge The Shamrock Organization Restructuring Strategic Stretch and Leverage The Post-Modern Organization The Cyclical Organization The Spider-Web Organization The Post-Industrial Organization The Turbulent Organization The Chaotic Organization

Most management theories concerning change and stability are in their infancy. Most theories still focus on change management, with little reference to the need for, or benefits of, organisational, stability [8]. The most successful organisations are the ones that value both stability and change, and try to balance the divergent practices. Even though change costs money, it is the stability that earns money. Successful organisations, such as Sony and GE have an organisational culture that promotes stability, but at the same time stays innovative by institutionalising change.

Change should be seen as a journey, not a blueprint, where change is non-linear, loaded with uncertainty and excitement, and produces sometimes perverse outcomes. Personal change needs to precede organisational change and acceptance requires a change in attitude. The more complex the change, the less we are able to force it. We should stop focusing on the individual parts but try to see the issues in totality. The issues/challenges we face should not be seen as attempts at problem-solving, because problem-solving is reactive and often functions as a way of maintaining the status quo rather than enabling fundamental change. Labelling an issue/challenge as a “problem” allows us to distance ourselves from the “problem”, which subsequently inhibits our ability to see the true situation. Instead, we should view challenges as learning opportunities.

The toughest part, though, is to learn to love ambiguity: simultaneously pushing for change while allowing self-learning to unfold and being prepared for a journey of uncertainty. If we can simply allow ourselves to be comfortable with all the seemingly unrelated bits and pieces of information – most of which are contradictory, ill-fitting and plain confusing – we can discover new ways to understand a situation which can eventually emerge.

At the same time, we also need to create a personal vision but not be blinded by it, while focusing on what we can do as individuals rather than on what we can’t do. It is also helpful to develop a perspective of looking at problems/challenges as sources of creative resolution with a willingness to learn and develop. Other measures we can take are: to try to value the individual and the group, incorporate centralising and decentralising forces, and be internally cohesive but externally oriented [9]. Change is too important a task to be left entirely to the experts and the individuals in the change process must be actively involved in its execution to achieve the desired outcomes.

The long-term costs of failed change efforts include lost time, energy, revenues, employees, increased cynicism, depression, anger, fear, increased resistance to change and misperceptions about change management. Therefore, it is vital that organisations desiring change must be committed to the efforts, otherwise the result of a failed effort to change is an organisation that is worse off than when it first started.

"The more things change, the more they are the same." [10]
